# Role for serotonin2A (5-HT2A) and 2C (5-HT2C) receptors in experimental absence seizures

**DOI:** 10.1016/j.neuropharm.2016.04.016

**Published:** 2016-09

**Authors:** Marcello Venzi, François David, Joachim Bellet, Anna Cavaccini, Cristiano Bombardi, Vincenzo Crunelli, Giuseppe Di Giovanni

**Affiliations:** a Neuroscience Division, School of Bioscience, Cardiff University, Museum Avenue, Cardiff CF10 3AX, UK; b University of Bologna, Department of Veterinary Medical Sciences, Bologna, Italy; c Department of Physiology and Biochemistry, University of Malta, Malta; d Werner Reichardt Centre for Integrative Neuroscience, Tuebingen University, Tuebingen, Germany

**Keywords:** Absence epilepsy, Selective serotonin 2 receptor drugs, EEG, Serotonin, (5-HT), 5-HT receptor, (5-HTR), Knockout, (KO), Meta-chlorophenylpiperazine, (mCPP), Maximal dentate gyrus activation, (MDA), Absence seizures, (ASs), Genetic absence epilepsy rat from Strasbourg, (GAERS), Spike-and-wave discharges, (SWDs), Intraperitoneally, (i.p.), Two-way analysis of variance, (ANOVA), Wistar Albino Glaxo/Rij, (WAG/Rij), Thalamic reticular nucleus, (NRT), Rapid eye movement, (REM), Cannabinoid, (CB)

## Abstract

Absence seizures (ASs) are the hallmark of childhood/juvenile absence epilepsy. Monotherapy with first-line anti-absence drugs only controls ASs in 50% of patients, indicating the need for novel therapeutic targets. Since serotonin family-2 receptors (5-HT_2_Rs) are known to modulate neuronal activity in the cortico-thalamo-cortical loop, the main network involved in AS generation, we investigated the effect of selective 5-HT_2A_R and 5-HT_2C_R ligands on ASs in the Genetic Absence Epilepsy Rats from Strasbourg (GAERS), a well established polygenic rat model of these non-convulsive seizures. GAERS rats were implanted with fronto-parietal EEG electrodes under general anesthesia, and their ASs were later recorded under freely moving conditions before and after intraperitoneal administration of various 5-HT_2A_R and 5-HT_2C_R ligands. The 5-HT_2A_ agonist TCB-2 dose-dependently decreased the total time spent in ASs, an effect that was blocked by the selective 5-HT_2A_ antagonist MDL11,939. Both MDL11,939 and another selective 5-HT_2A_ antagonist (M100,907) increased the length of individual seizures when injected alone. The 5-HT_2C_ agonists lorcaserin and CP-809,101 dose-dependently suppressed ASs, an effect blocked by the selective 5-HT_2C_ antagonist SB 242984. In summary, 5-HT_2A_Rs and 5-HT_2C_Rs negatively control the expression of experimental ASs, indicating that selective agonists at these 5-HT_2_R subtypes might be potential novel anti-absence drugs.

## Highlights

• 5-HT2AR activation decreases absence seizures in GAERS.
• 5-HT2CR activation decreases absence seizures in GAERS.
• 5-HT2AR blockade increases absence seizures in GAERS.
• 5-HT2CR blockade does not affect absence seizures in GAERS.

## 1 Introduction

Since the original suggestion in the late 1950s' (Bonnycastle et al., 1957), many studies have supported the idea that the serotonin (5-HT) system might be implicated in both focal and generalized epilepsy. In particular, it has been shown that an increase in 5-HT tone is associated with an increased seizure threshold (and/or antiepileptic activity), whilst a reduced seizure threshold follows a decrease in 5-HT levels (reviewed in (Bagdy et al., 2007)). Moreover, many anti-epileptic drugs enhance brain extracellular 5-HT levels and many selective serotonin reuptake inhibitors (SSRIs) show an antiepileptic effect (Bagdy et al., 2007). Despite this large body of evidence, none of the currently available anti-epileptic drugs preferentially targets the 5-HT system, probably because of the lack of selective/specific ligands, the presence of harmful off-target effects and the complexity of the 5-HT receptor (5-HTR) system and its signaling pathways (Hannon and Hoyer, 2008; Stroth and Svenningsson, 2012).

The current classification of 5-HTRs comprises up to 14 subtypes and the generation of selective pharmacological and genetic tools, i.e., knockout (KO) mice, to investigate the contribution of individual receptors is fairly recent (Hannon and Hoyer, 2008). Among the different 5-HTR subtypes (Hoyer et al., 2002), many lines of evidence suggest an involvement of 5-HT_2_Rs in seizures (reviewed in (Di Giovanni and De Deurwaerdère, 2016, Guiard and Di Giovanni, 2015, Isaac, 2005, Jakus and Bagdy, 2011)). 5-HT_2C_R KO mice display spontaneous tonic-clonic seizures which are occasionally lethal (Tecott et al., 1995), and a decreased threshold for various convulsing stimuli, e.g., kindling, pentylenetetrazol (PTZ), electroshock, audiogenic stimuli (Applegate and Tecott, 1998, Heisler et al., 1998). Further evidence of the protective role for 5-HT_2C_Rs against convulsive seizures comes from experiments using non-selective 5-HT_2C_ agonists which raise the threshold for PTZ- and electroshock-induced seizures (Upton et al., 1998). On the other hand, some 5-HT_2C_R agonists with different pharmacological profiles, i.e., meta-chlorophenylpiperazine (mCPP) and lorcaserin, but not RO60-0175 (Martin et al., 1998) are able to stop the elongation of the electrically triggered hippocampal maximal dentate gyrus activation (MDA) in a limbic seizure model (Orban et al., 2014). As for 5-HT_2A_Rs, fewer studies have investigated the role of these receptors in seizures, with most evidence showing that their activation has an antiepileptic effect (Gharedaghi et al., 2014, Guiard and Di Giovanni, 2015).

The evidence of a role for 5-HT_2_Rs in generalized non-convulsive seizures is more limited and the interpretation of these studies is hampered by the use of relatively unselective drugs (Bagdy et al., 2007, Di Giovanni and De Deurwaerdère, 2016, Guiard and Di Giovanni, 2015). In the groggy model of absence seizures (ASs) (Tokuda et al., 2007), the 5-HT_2A/2C_ mixed agonist DOI dose-dependently reduces ASs, an effect that is blocked by the non-selective 5-HT_2_R antagonist ritanserin (Ohno et al., 2010). In contrast, in the AY-9944 model of atypical ASs, mCPP has no effect, DOI dose-dependently decreases ASs and the moderately selective 5-HT_2A_R antagonist ketanserin increases ASs in a non-dose-dependent manner (Bercovici et al., 2006). These authors concluded that 5-HT_2A_Rs were responsible for this effect, although no 5-HT_2A_R antagonist was tested against the anti-absence action of DOI. As far as typical ASs are concerned, experiments in the Wistar Albino Glaxo/Rijswijk (WAG/Rij) rats, one of the best characterized rat models of these type of non-convulsive seizures (Coenen and Van Luijtelaar, 2003), have found that mCPP decreases ASs via 5-HT_2C_Rs (Jakus et al., 2003). Moreover, SB-242084 a selective 5-HT_2C_R antagonist, has no effect on ASs when administered alone, suggesting that 5-HT_2C_Rs do not play a tonic modulatory role (Jakus and Bagdy, 2011, Jakus et al., 2003). In the other well characterized rat model of typical ASs, the Genetic Absence Epilepsy Rat from Strasbourg (GAERS) (Danober et al., 1998) the contribution of the 5-HT system to ASs has only been partly investigated, probably because of the early negative results obtained with broad-spectrum first-generation 5-HTR agonists and antagonists or following modulation of the 5-HT tone by 5-HT uptake blockers and precursors (Marescaux et al., 1992a, Marescaux et al., 1992b) (reviewed in (Danober et al., 1998)).

In the present study, we evaluated the effects of pharmacological manipulation of 5-HT_2_Rs on typical ASs and the interictal EEG in GAERS using drugs selective for 5-HT_2A_Rs and 5-HT_2C_Rs. The potent 5-HT_2A_R agonist TCB-2 (McLean et al., 2006) was used in combination with the selective 5-HT_2A_R antagonists MDL11,939 (Dudley et al., 1988) and M100,907 (Table 1) (Kehne et al., 1996). As far as 5-HT_2C_Rs were concerned, we used CP-809,101, which shows ∼1000 fold selectivity for the 5-HT_2C_R over 5-HT_2A_R and represents the most selective 5-HT_2C_R drug currently available (Siuciak et al., 2007), and SB-242084, the most selective 5-HT_2C_R antagonist synthetized to date (Di Matteo et al., 2000, Kennett et al., 1997) (Table 1). Moreover, the anti-absence action of lorcaserin (Thomsen et al., 2008) was also investigated because, although it has only an approximately 10-fold higher affinity for 5-HT_2C_Rs compared to 5-HT_2A_Rs (Table 1), it is the first-in-class 5-HT_2C_R agonist available for human use (FDA, 2012) and has shown an antiepileptic profile in an animal model of temporal lobe epilepsy (Orban et al., 2014). Our results show that both 5-HT_2A_Rs and 5-HT_2C_Rs negatively control the expression of experimental ASs, suggesting that selective agonists at these 5HT_2_R subtypes might be potential novel anti-absence drugs.

## 2 Methods

Male GAERS rats (3–5 months old) were obtained from a colony bred at Cardiff University. Animals were housed in a 12:12 light cycle (lights on at 10.00 p.m. and off at 10.00 a.m.). All animal procedures were approved by the UK Home Office and carried out in accordance with Cardiff University ethical guidelines and in conformity with international law and policies (EU Directive, 2010/63/EU for animal experiments, ARRIVE guidelines and the Basel declaration including the 3R concept). All efforts were made to minimize animal suffering and to reduce the number of animals used (Lidster et al., 2015).

### 2.1 Surgery and EEG recordings

GAERS underwent chronic electrode implantation under general anesthesia (2% isofluorane). Epidural EEG electrodes (gold plated screws, Svenska Dentorama AB, Sweden) were implanted bilaterally over the frontal cortex, parietal cortex and in the cerebellum, as previously described (Cope et al., 2009). Animals were allowed to recover for at least 5 days. At 10.00 a.m. on the day of the experiment, the animals were placed into individual Plexiglas cages with access to food and water, and connected to a pre-amplifier (0.08 Hz high-pass filter, impedance 10 MΩ) and in turn to an analogue EEG amplifier (4-channel BioAmp, SuperTech Inc., Hungary) (1000 gain, low-pass filter at 500 Hz). The signal was digitized at 1000 Hz with a Cambridge Electronic Design (CED) Micro3 D.130 digitizer using CED Spike2 v7.3.

### 2.2 Experimental protocol

Once GAERS had been connected to the recording apparatus, they were left undisturbed for 1 h (habituation period). After that, video and EEG recordings commenced and continued for 40 min (control period). If the experiment involved pre-treatment of a 5-HT_2A/2C_ antagonist, the antagonist (or the corresponding vehicle) was intraperitoneally (i.p.) injected 10 min before the end of the control period. At the end of the control period, the animal was injected (i.p.) with the 5-HT drug of interest (or corresponding vehicle) and video and EEG recordings continued for the subsequent 2 h (treatment period). Drug injection order and doses were randomized in an incomplete crossover design and each drug-naïve animal received a maximum of three treatments in increasing dose protocols (vehicle, low dose, high dose) or four treatments when testing one dose of an agonist vs antagonist (e.g. vehicle + vehicle, vehicle + agonist, antagonist + vehicle, antagonist + agonist). The washout period was 5 days.

### 2.3 ASs detection and quantification

The detection of spike-and-wave discharges (SWDs) was semi-automatic, aided by the SeizureDetect script (kindly provided by Steve Clifford, CED) in Spike2 v7.3 (CED, UK), designed to discriminate between sleep spindles and ASs. The data was high pass-filtered (DC remove, time constant 0.1 s) and the user selected manually a interictal segment of control, awake desynchronized EEG. The script automatically detected the crossings of a threshold defined as 5–9 SD above and below the mean voltage of the manually selected interictal period. Subsequently the crossings above threshold were grouped together in order to define putative SWDs according to 4 parameters. In order to define the start of the putative SWD, two crossings needed to be separated by less than the max onset interval (0.2 s). To be incorporated into the putative SWD the timing of the following crossings had to be lower than the maximum continuation interval (0.4 s). Putative SWDs less than 0.5 s apart were amalgamated. Putative SWDs with a duration of less than 1 s were discarded. The interval of consecutive crossings in each putative SWD was then used to define instantaneous frequencies (in the time domain). Only putative SWDs which had ≥75% of their peaks in the 5–12 Hz frequency range were selected, resulting in the exclusion sleep/drowsiness epochs and artifacts. This semi-automatic SWD selection was further refined by visual inspection with a custom made Matlab script (Matlab R2013b, The Mathworks Inc., USA) and confirmed by the concomitant presence of behavioral arrest in the video recordings (Depaulis et al., 2016). Three parameters of ASs were quantified: total time spent in ASs, average duration of single ASs and total number of ASs (Marescaux et al., 1992a). For each animal and dose, the quantification of ASs was done in 20 min epochs and the value of each of the three parameters during the treatment period was normalized by expressing it as a percentage of the corresponding parameter in the control period. All statistical tests were performed on the data following this normalization (see section Statistical Analysis). For clarity, data are presented in the figures as percentage of the corresponding vehicle group (100% dotted line in figures).

### 2.4 Ictal EEG analysis

Spectral analysis of previously detected seizures was performed in order to determine the peak frequency of SWDs and to investigate possible changes induced by the drug treatment. Briefly, continuous wavelet transform (Morlet mother wavelet, f0 = 1, range 5–14 Hz) was employed to determine the time-frequency profile of SWDs using a Matlab script kindly provided by Dr Dmytro Iatsenko (Iatsenko et al., 2013) (freely available at http://www.physics.lancs.ac.uk/research/nbmphysics/diats/tfr/). The instantaneous frequencies corresponding to each previously detected SWD during the treatment period were extracted from the wavelet power maxima in the range 5–14 Hz. The mean frequency was calculated for each SWD and then averaged across all SWDs for each animal.

### 2.5 Interictal EEG analysis

Spectral analysis of the interictal EEG was performed on the raw data after preprocessing with Fieldtrip (Oostenveld et al., 2011). This involved resampling the data to 200 Hz after applying an anti-aliasing (low-pass) FIR filter. Each EEG recording was analyzed blind and artifacts and sleep epochs were marked manually with custom-made Matlab scripts. Time-frequency decomposition was performed with a script kindly provided by Dmytro Iatsenko (Leicester University, UK) performing short-time Fourier transform (binning 1–80 Hz, Gaussian window, f0 = 1) on 20-min bins, matching those used for AS analysis. Samples corresponding to previously detected artifacts, SWDs and sleep periods were excluded from the analysis. Power spectra were then averaged over time for each 20-min epoch. Five EEG bands were defined for the analysis: delta (1–4 Hz), theta (5–8 Hz), alpha (9–12 Hz), beta (13–30 Hz) and gamma (31–80 Hz). Data within 48–52 Hz were excluded from the analysis of the gamma band to avoid contamination with the 50 Hz power line.

### 2.6 Statistical analysis

All statistical analyses were performed with Graphpad Prism version 5.00 (GraphPad Software, San Diego, USA). The effect of drug administration on the expression of absence seizures was analyzed via non-repeated measures two-way analysis of variance (ANOVA) with drug and time as factors. Dunnet's post-hoc testing was employed to test for the simple main effect of drug vs. vehicle for the full treatment period, whereas Sidak's multiple comparison test was applied to analyze the time-course of the drug effects for each 20 min bin compared to the corresponding time for vehicle. Statistical analysis on peak SWD frequency was performed using unpaired *t*-test. The effect of drug administration on the interictal EEG was analyzed via non-repeated measures two-way analysis of variance (ANOVA) with the frequency bands and time as factors. Sidak's multiple comparison test was applied to analyze the time-course of the drug effect on the interictal EEG for each 20 min bin compared to the corresponding time for vehicle. All quantitative data are reported in the test and figures as mean ± SEM (unless otherwise stated).

### 2.7 Drugs

The selective 5-HT_2A_R antagonist M100907 (Kehne et al., 1996) was purchased from Sigma-Aldrich (UK). The selective 5-HT_2C_R antagonist SB-242084 (Kennett et al., 1997), the potent 5-HT_2A_R agonist TCB-2 (McLean et al., 2006), the selective 5-HT_2A_R antagonist MDL11,939 (Dudley et al., 1988) and the selective 5-HT_2C_R agonist CP-809,101 (Siuciak et al., 2007) were purchased from Tocris Biosciences (UK). The 5-HT_2C_R agonist Lorcaserin (Thomsen et al., 2008) was a kind gift from Arena Pharmaceuticals Inc. (USA). All common laboratory reagents were purchased from Sigma-Aldrich (UK). TCB-2 and lorcaserin were dissolved in 0.9% saline. CP-809,101 was dissolved in 2% Tween 80. SB-242084 and M100907 were dissolved in 25 mM citric acid, 8% (2-Hydroxypropyl)-β-cyclodextrin (w/v) in 0.9% saline. MDL11,939 was dissolved in 0.9% saline and 5% glacial acetic acid and the pH adjusted to 7.0 with NaOH. Drugs were selected for being the most selective 5-HT_2A_R/5-HT_2C_R agents available to date for research use (Table 1) and, in the case of Lorcaserin, in view of its clinical use (FDA, 2012). TCB-2 was selected for its high affinity for 5-HT_2A_R because, to the best of our knowledge, no selective 5-HT_2A_R agonist has been synthesized to date.

## 3 Results

The behavioral and EEG features of ASs recorded in vehicle-treated, freely moving GAERS were similar to those previously reported for this experimental model under similar experimental conditions (Cope et al., 2009, Danober et al., 1998). These included behavioral arrest, with occasional head and vibrissae twitching, and SWDs of 16.1 ± 14.3 s duration and 6.90 ± 0.72 Hz peak frequency (n = 8914 seizures, mean ± standard deviation) (see Fig. 3A).

### 3.1 Effect of 5-HT_2A_R modulation on spontaneous ASs

**Fig. 1:**
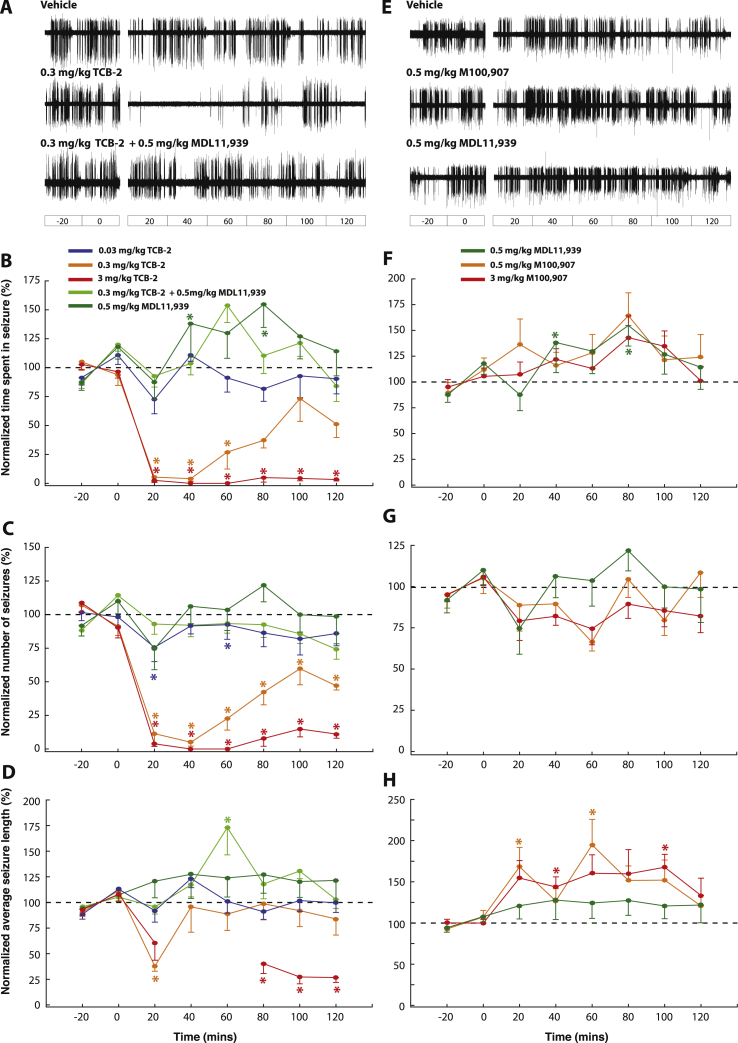
Effects of the 5-HT_2A_R agonist TCB-2 and the 5-HT_2A_R antagonists MDL11,939 and M100,907 on ASs. (A) Representative EEG traces for GAERS injected i.p. with vehicle, 0.3 mg/kg TCB-2 and 0.3 mg/kg TCB-2 following pre-treatment with MDL11,939 (0.5 mg/kg, i.p.) (interruption in each trace indicates time of injection). Dose response curves for TCB-2 effects on normalized total time spent in seizures (B), number of seizures (C) and seizure length (D). Pre-treatment with the selective 5-HT_2A_R antagonist MDL11,939 (0.5 mg/kg) blocked the effect of TCB-2 (3 mg/kg) on the three seizure parameters. (E) Representative EEG traces for GAERS injected i.p. with vehicle, 0.5 mg/kg MDL11,939 and 0.5 mg/kg M100,907 (interruption in each trace indicates time of injection). Effect of MDL11,939 (0.5 mg/kg) and M100,907 (0.5–3 mg/kg) on normalized total time spent in seizures (F), seizure length (G) and number of seizures (H). Although no single time point reached significance after post-hoc testing, both 5-HT_2A_R antagonists significantly increased the time spent in seizures and seizure length when considering the overall treatment time (see text for details). All values are normalized to the control period (−40 to 0 min), and are expressed as a percentage of their respective vehicle group for clarity. Values represent mean ± SEM. Time zero indicates the time of injection of the agonist, while the antagonist MDL11,939 was injected 10 min before TCB-2 administration. Asterisks indicate p < 0.05 for a given time bin in the treatment group vs the corresponding time bin in the vehicle group (two-way ANOVA, Sidak's multiple comparison test). TCB-2 (0.03–3 mg/kg): n = 9–6; TCB-2 (0.3 mg/kg) ± MDL11,939 (0.5 mg/kg): n = 7; M100,907 (0.5–3 mg/kg): n = 11–9; MDL11,939 (0.5 mg/kg): n = 11.

GAERS were injected i.p. with vehicle (n = 9) or TCB-2 (0.03, 0.3, 3 mg/kg; n = 6–9 for each dose) in a randomized order, and the resulting effects (normalized to the effect of the vehicle group) are shown in Fig. 1A–D. As revealed by post-hoc testing for the simple main effect of the drug, TCB-2 decreased the total time spent in seizure compared to vehicle (0.03 mg/kg: 10.1 ± 4.6% overall decrease, p < 0.05; 0.3 mg/kg: 69.4 ± 9.2% overall decrease, p < 0.001; 3 mg/kg: 97.4 ± 1.5% overall decrease, p < 0.001). This effect was dose-dependent (0.03 vs 0.3 mg/kg: p < 0.001; 0.3 vs 3 mg/kg: p < 0.001) (Fig. 1B). The average number of seizures was also significantly decreased by all doses of TCB-2 (0.03 mg/kg: 14.4 ± 9.7% overall decrease, p < 0.001; 0.3 mg/kg: 68.6 ± 7.2% overall decrease, p < 0.001; 3 mg/kg: 93.7 ± 2.8% overall decrease, p < 0.001), an effect that was dose-dependent (0.03 vs 0.3 mg/kg: p < 0.001; 0.3 vs 3 mg/kg: p < 0.001) (Fig. 1C). There was no effect on the length of individual ASs at 0.03, while 0.3 mg/kg TCB-2 elicited a reduction in seizure length at 20 min post-injection (62.2 ± 4.9% decrease, p < 0.001). Moreover 3 mg/kg TCB-2 elicited a drastic reduction of seizure length (81.1 ± 7.0% decrease at 80–120 min, p < 0.001) (Fig. 1D). Note that during the 40 and 60 min post-injection time-bins of 3 mg/kg TCB-2 no seizures were observed, and thus no estimation of seizure length was possible (gaps in Fig. 1D). Finally, an increase of the SWD peak frequency was observed only at the highest dose of TCB-2 (vehicle: 7.1 ± 0.1 Hz; 3 mg/kg TCB-2: 7.6 ± 0.2 Hz; p < 0.05) (Fig. 3B).

Pre-treatment with the 5-HT_2A_ antagonist MDL11,939 (0.5 mg/kg, i.p., n = 7) blocked the effect of 0.3 mg/kg TCB-2 on the total time spent in seizure (Fig. 1A, B) and seizure length (Fig. 1D) (simple main effect of 0.5 mg/kg MDL11,939 + 0.3 mg/kg TCB-2 vs vehicle, ns), and greatly attenuated the effect of the agonist on the number of seizures (Fig. 1C) (simple main effect of MDL11,939 + TCB-2 vs vehicle, 11.5 ± 7.9% overall decrease, p < 0.05; compared to 68.6 ± 7.2% overall decrease for 0.3 mg/kg TCB-2 vs vehicle). Interestingly, MDL11,939 (0.5 mg/kg, n = 11) on its own significantly increased the total time spent in ASs compared to vehicle (overall 25.5 ± 21.1% increase, p < 0.05). Moreover, a significant effect of this drug was observed on seizure length (overall 23.5 ± 18.9% increase, p < 0.05), but not on seizure number. To further confirm these effects of MDL11,939, another selective 5-HT_2A_R antagonist, M100,907, was injected in a naïve group of GAERS (Fig. 1E–H). Post-hoc testing on the simple main effect of the drug showed that M100,907 induced a significant increase in the total time spent in ASs both at 0.5 mg/kg (31.7 ± 20.4% overall increase, p < 0.001, n = 11) and at 3 mg/kg (20.1 ± 14.6% overall increase, p < 0.05, n = 9). This effect was driven by a significant increase in the seizure length, both at 0.5 mg/kg (52.3 ± 20.7% overall increase, p < 0.001) and at 3 mg/kg (53.2 ± 20.3% overall increase, p < 0.001). In addition, M100,907 induced a small, but significant, decrease in the number of ASs both at 0.5 mg/kg (10.4 ± 10.7% overall decrease, p < 0.01) and at 3 mg/kg (17.9 ± 9.2% overall decrease, p < 0.01). Moreover, while no significant change of the peak SWD frequency was found upon treatment with MDL11,939, both doses of M100,907 induced a modest, but significant decrease in the SWD peak frequency (vehicle: 7.1 ± 0.1 Hz vs 0.5 mg/kg: 6.85 ± 0.05 Hz; p < 0.05; vs 3 mg/kg: 6.9 ± 0.05; p < 0.05) (Fig. 3B).

The block of ASs by TCB-2 was accompanied by behavioral components typical of 5-HT_2A_R activation in rodents (e.g., wet dog shakes, head twitches) (Bedard and Pycock, 1977), that were short-lasting compared to its antiabsence effect. Importantly, the effect of TCB-2 on both ASs and the animal behavior was blocked by pretreatment with the 5-HT_2A_ antagonist MDL11,939, confirming that the effect was indeed driven by the activation of 5-HT_2A_Rs. In summary, these results indicate that selective activation of 5-HT_2A_Rs markedly decreases spontaneous, genetically determined ASs and that this 5-HTR subtype exerts a negative tonic modulation of these non-convulsive seizures.

### 3.2 Effect of 5-HT_2C_R modulation on spontaneous ASs

**Fig. 2:**
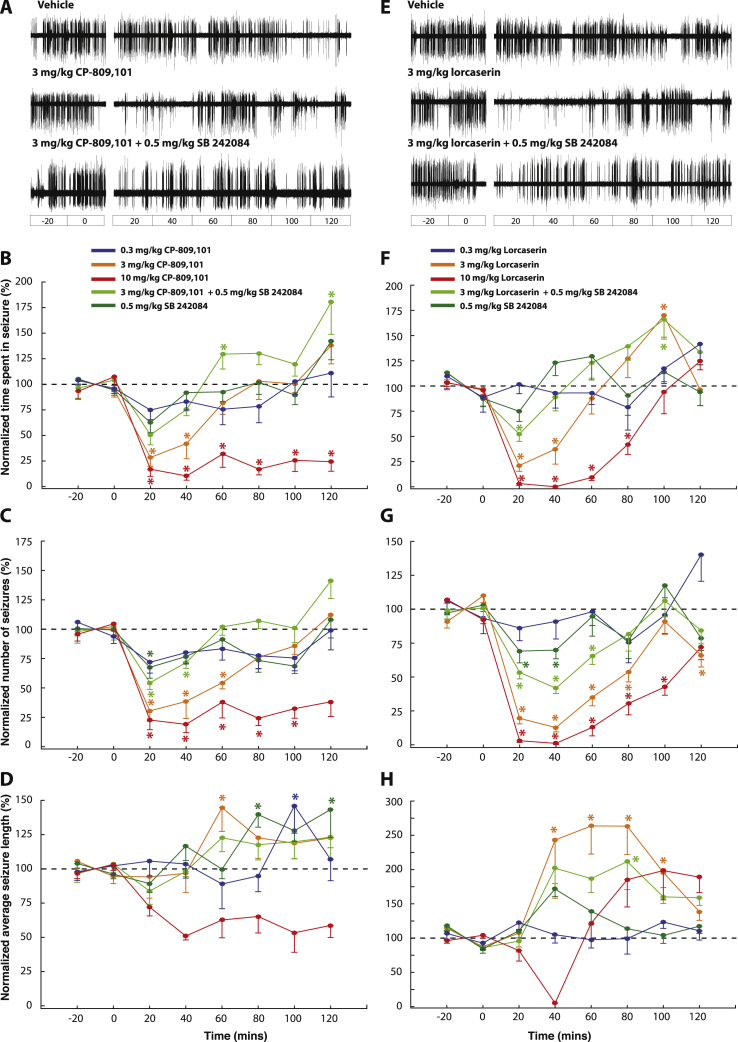
The 5-HT_2C_R agonists lorcaserin and CP-809,101 dose-dependently decrease ASs. (A) Representative EEG traces for GAERS injected i.p. with vehicle, 3 mg/kg CP-809,101 and 3 mg/kg CP-809,101 following pre-treatment with SB242084 (0.5 mg/kg, i.p.) (interruption in each trace indicates time of injection). Dose response curve of CP-809,101 at 0.3-3-10 mg/kg for normalized total time spent in seizure (B), number of seizures (C) and seizure length (D). Pre-treatment with the selective 5-HT_2C_R antagonist SB2402084 (0.5 mg/kg) partially blocked the effect of CP-809,101 (3 mg/kg) on the three seizures parameters. (E) Representative EEG traces for GAERS injected i.p. with vehicle, 3 mg/kg lorcaserin and 3 mg/kg lorcaserin following pre-treatment with SB242084 (0.5 mg/kg, i.p.) (interruption in each trace indicates time of injection). Dose response curves of lorcaserin (right side of the figure) at 0.3-3-10 mg/kg for normalized total time spent in seizure (F), number of seizures (G) and seizure length (H). Pre-treatment with the selective 5-HT_2C_R antagonist SB2402084 (0.5 mg/kg) partially blocked the effect of CP-809,101 (3 mg/kg) on the three seizures parameters. All values are normalized to the control period (−40 to 0 min), and are expressed as a percentage of their respective vehicle group for clarity. Values represent mean ± SEM. Time zero indicates the time of injection of the agonist, while the antagonist SB2402084 was injected 10 min before the agonist administration. Asterisks indicates p < 0.05 for a given time bin in the treatment group vs the corresponding time bin in the vehicle group (two-way ANOVA, Sidak's multiple comparison test). CP-809,101 (0.3–10 mg/kg): n = 10–6; CP-809,101(3 mg/kg) ± SB242084 (0.5 mg/kg): n = 10; lorcaserin (0.3–10 mg/kg): n = 8–6; lorcaserin (3 mg/kg) ± SB242084 (0.5 mg/kg): n = 9.

**Fig. 3:**
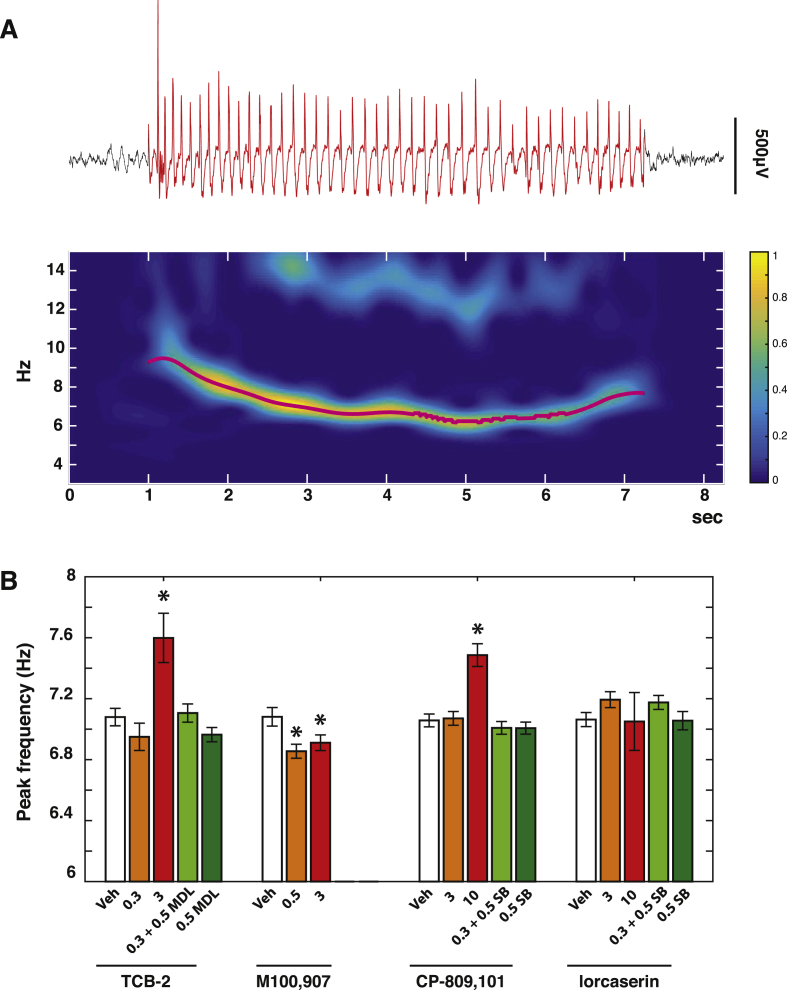
**Effect 5-HT**_**2A/C**_**Rs modulation on SWDs peak frequency**. (A) Representative spike and wave discharge (SWD) (red, top) and corresponding wavelet power spectrum on a time-frequency representation (bottom). The peak frequency of the SWD was identified by extracting the instantaneous frequency of the power maximum in the range 5–14 Hz at each time point of the SWD (magenta line in time-frequency plot) and subsequently calculating its mean. (B) Changes (mean ± SEM) in SWD peak frequency induced by administration of 5-HT_2A_R and 5-HT_2C_R agonists and antagonists (bottom row) isolated or in combination with another drug (doses in mg/kg are reported at the bottom of the graph). Asterisks indicate p < 0.05 (independent samples *t*-test for drug vs vehicle).

We next investigated the action of the selective 5-HT_2C_R agonist CP-809,101 in different groups of GAERS at doses (0.3, 3 and 10 mg/kg, n = 6–10 per dose) similar to those used in previous studies (Higgins et al., 2013b, Siuciak et al., 2007). No effect of 0.3 mg/kg was found on the total time spent in ASs, seizure length and number of seizures (Fig. 2A–D). However CP-809,101 induced a decrease in the total time spent in seizure at 3 mg/kg (17.7 ± 13.1% overall reduction, p < 0.001) and 10 mg/kg (78.9 ± 8.3% overall reduction, p < 0.001). The effect of 3 mg/kg was strong but of short duration, with post-hoc testing showing a significant effect for the first 40 min post-injection (64.7 ± 11.7% decrease, p < 0.05), whereas for 10 mg/kg the total time spent in ASs was drastically reduced throughout the 2 h treatment period (Fig. 2B). Moreover, whereas 3 mg/kg CP-809,101 increased seizures length (21.4 ± 14.4% overall increase, p < 0.05), 10 mg/kg elicited a significant reduction in seizure length (39.5 ± 9.5% overall reduction, p < 0.001) (Fig. 2D). The number of seizures was reduced for both 3 and 10 mg/kg CP-809,101, an effect that was significant for 60 min (58.7 ± 9.5% mean reduction, p < 0.05) and 100 min post-injection (72.7 ± 8.6% mean reduction, p < 0.05) (Fig. 2C). Pre-treatment with 0.5 mg/kg SB-242084 (n = 10) blocked the effect of CP-809,101 (3 mg/kg) on the total time spent in ASs (simple main effect of 0.5 mg/kg SB-242084 + 3 mg/kg CP-809,101 vs vehicle, ns), but only partially blocked the effect of this agonist on seizure length and number of seizures (Fig. 2A–D). Moreover, a significant increase of the SWD peak frequency was observed following the highest dose of CP-809,101 (vehicle: 7.1 ± 0.1 Hz, 10 mg/kg: 7.5 ± 0.1 Hz; p < 0.05) (Fig. 3B).

The action of the less selective 5-HT_2C_ agonist lorcaserin (Thomsen et al., 2008) (0.3, 3, 10 mg/kg; n = 6–10 per dose) was more complex than that observed following CP-809,101 treatment. At 0.3 mg/kg lorcaserin had no significant effect on total time spent in ASs, seizure length and number of seizures (Fig. 2F, G, H). Although the overall effect of 3 mg/kg lorcaserin was only a small but significant decrease in the total time spent in ASs (10.1 ± 15.2%, p < 0.05), post-hoc testing showed that this action was due to clear time-dependent biphasic effect, i.e. a marked decrease in total AS time during the first 40 min post-injection (20 min: 78.9 ± 5.7% p < 0.001; 40 min; 62.6 ± 14.9%, p < 0.01), which was followed by an increase 100 min post-injection (69.8 ± 21.5; p < 0.01) (Fig. 2F). This complex action of 3 mg/kg lorcaserin on total AS time could be explained by the different effect that this dose had on the number of seizures and seizure length, with the former markedly decreasing for almost all time points post-injection (overall reduction: 53.6 ± 6.5%, p < 0.001) (Fig. 2G) while the latter was increased during the same observation period (overall enhancement: 201 ± 30%, p < 0.001) (Fig. 2H). The higher dose of lorcaserin (10 mg/kg) elicited an overall more potent reduction in the total time spent in ASs (overall decrease: 54.5 ± 7.5%, p < 0.001) which could also be explained by a marked reduction in the number of seizures. A trend for an increase in seizure length was evident in the second hour of the recording although no individual time bin reached significance in the post-hoc testing (Fig. 2H). Moreover, we observed no change in the SWD peak frequency following any doses of locarserin (Fig. 3B).

Pre-treatment with the selective 5-HT_2C_ antagonist SB 242084 (Di Matteo et al., 2000) (0.5 mg/kg, n = 9) almost fully abolished the effect of lorcaserin (3 mg/kg) on the total time spent in ASs (simple main effect of SB 242084 + lorcaserin vs vehicle, ns), but only partially blocked the effect of the agonist on seizure length and number of seizures (Fig. 2F–H). SB 242084 injected alone (n = 10) had no significant effects on the total time spent in seizures and on seizure length. Surprisingly, SB 242084 decreased the number of seizures in the first 20 and 40 min post-injection (20 min: 30.1 ± 8.7% p < 0.01; 40 min; 30.1 ± 6.4%, p < 0.05) (Fig. 2F–H). No change in the SWD peak frequency was observed following treatment with SB 242084 (Fig. 3B).

Both CP-809,101 and locarserin produced 5-HT_2C_ behavioral effects consistent with those reported previously (Di Giovanni and De Deurwaerdère, 2016). In particular penile grooming was observed at all doses of both 5-HT_2C_ agonists, and hypolocomotion was evident especially at high doses (i.e., 10 mg/kg lorcaserin and CP-809,101). These effects were short-lasting compared to their antiabsence effect and antagonized by pre-treatment with the selective 5-HT_2C_ antagonist SB 242084, which had no behavioral effect on his own, consistent with previous reports (Higgins et al., 2001, Kennett et al., 1997). In summary, these results indicate that selective activation of 5-HT_2C_Rs decreases spontaneous, genetically determined ASs and that this 5-HTR subtype does not appear to exert a tonic modulation on these non-convulsive seizures. Of note, SB 242084 showed an antiabsence effect at some time-points, confirming the complexity of the 5-HT_2C_R system (see (Di Giovanni and De Deurwaerdère, 2016)).

### 3.3 Effects of 5-HT_2A_R and 5-HT_2C_R agonists on interictal EEG

**Fig. 4:**
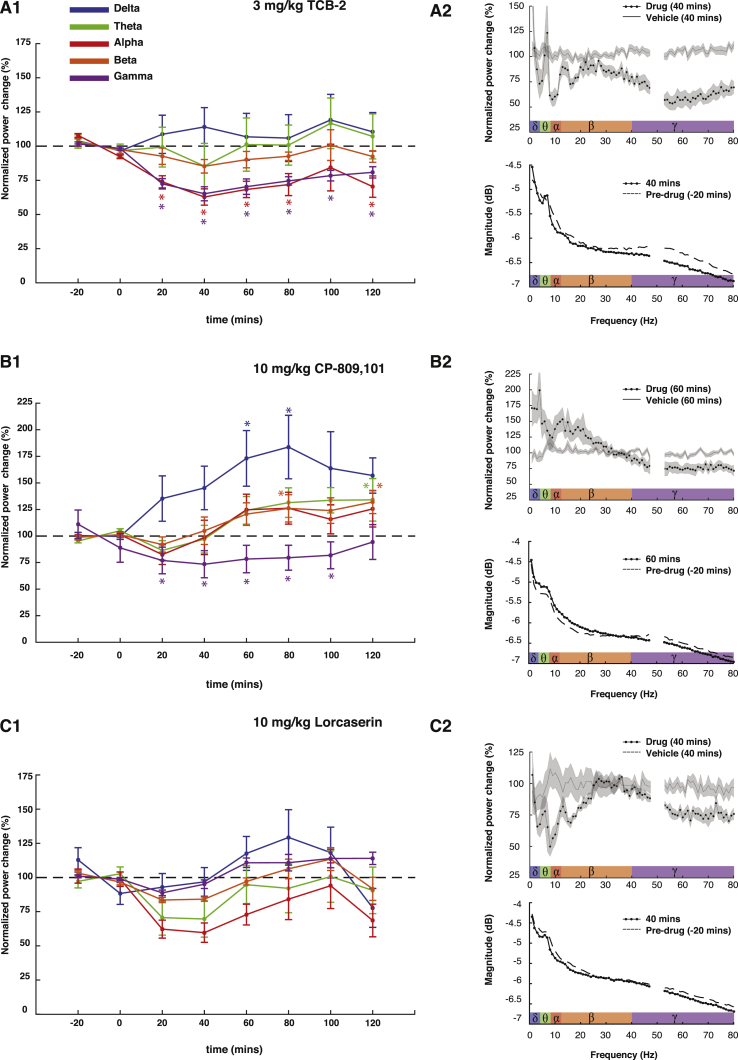
**Effect of TCB-2, CP-809,101 and lorcaserin on interictal EEG**. (A1) TCB-2 (3 mg/kg, n = 6) significantly reduced EEG power in the alpha (8–12 Hz) and gamma (30–80 Hz) bands compared to vehicle (n = 7). (A2) Mean normalized power change at 40 min compared to the relative time point in the vehicle injected animals (top) and raw EEG spectrum at 40 min compared to pre-drug (bottom). (B1) CP-809,101 (10 mg/kg, n = 6) significantly increased EEG power in the delta (1–4 Hz) and decreased power in the gamma band (30–80 Hz) compared to vehicle (n = 8). (B2) Mean normalized power change at 60 min compared to equivalent time point in the vehicle injected animals (top) and raw EEG spectrum at 60 min compared to pre-drug (bottom). (C1) In the lorcaserin treated animals (10 mg/kg) no individual point reached statistical significance after post-hoc testing, although a trend for an effect in the delta and gamma bands is visible at 40 min (*C*2). All values are normalized to the control period (−40 to 0 min), and are expressed as a percentage of their respective vehicle group. Values represent mean ± SEM. Asterisks indicate p < 0.05 for a given time bin in the treatment group vs the corresponding time bin in the vehicle group (two-way ANOVA, Sidak's multiple comparison test).

In addition to modifying ASs, both at the EEG and behavioral level, the highest doses of TCB-2 (3 mg/kg) and CP-809,101 (10 mg/kg) were also able to produce significant modifications of the power of different frequency bands in the interictal EEG (Fig. 4). TCB-2 induced a long-lasting decrease in the power of the gamma (26.4± 4.4% mean reduction, p < 0.001) and alpha (28.0 ± 8.5% mean reduction, p < 0.001 vs vehicle) frequency bands. CP-809,101 elicited a reduction in the gamma band power (26.1 ± 21.5%, mean decrease, p < 0.05) and a drastic increase in the delta band (64.5 ± 23.0% mean increase, p < 0.001). Finally, a trend for an increase in the delta band power and a decrease in the alpha band was apparent in the interictal EEG following the injection of 10 mg/kg lorcaserin although no individual time epoch reached statistical significance.

## 4 Discussion

The main conclusions of this study are twofold i) selective activation of both 5-HT_2A_Rs and 5-HT_2C_Rs decreases spontaneous, genetically determined ASs, and ii) only 5-HT_2A_Rs exert a negative tonic modulation on these non-convulsive seizures.

### 4.1 Effect of 5-HT_2A_R ligands on ASs

5-HT_2A_Rs have not previously been implicated in the pathogenesis or modulation of ASs, except from the indirect evidence provided by the block of ASs by DOI, a mixed 5-HT_2A/2C_ agonist, in the groggy rats, a model of these non-convulsive seizures that still remains to be fully characterized (Ohno et al., 2010). Regardless of the current lack of highly selective 5-HT_2A_ agonists (Nichols, 2004, Roth, 2011), the solidity of our results is supported by our approach of using, as in previous studies by other groups (Fox et al., 2010, Goda et al., 2013), a potent (i.e., nM affinity) 5-HT_2A_R agonist (TCB-2) in conjunction with a selective 5-HT_2A_ antagonists (MDL11,939) at a concentration that is known to block 5-HT_2A_- but not 5-HT_2C_-mediated behaviors (Fletcher et al., 2002).

The presence of an inhibitory tone of 5-HT_2A_Rs on ASs, as indicated by a significant increase in seizure length following administration of either of the two 5-HT_2A_R antagonists used in this study (MDL11,939 and M100,907), supports the idea that these receptors tonically affect the duration of seizures in GAERS. The conclusion that drugs blocking 5-HT_2A_Rs might increase AS duration is in line with the findings in WAG/Rij rats treated with atypical antipsychotics where risperidone but not quetiapine was found to increase AS duration (Citraro et al., 2015). Indeed, risperidone has a ∼100 fold higher affinity that quetiapine for 5-HT_2A_Rs (Richtand et al., 2007). However, it should be noted that atypical antipsychotics affect multiple neurotransmitter systems which may also underlie their effects on seizures.

### 4.2 Effects of 5-HT_2C_R ligands on ASs

Of the two 5-HT_2C_R agonists employed in this study, CP-809,101 displays a high (>1000 fold) selectivity for 5-HT_2C_Rs over 5-HT_2A_Rs whereas lorcaserin has a lower (∼10 fold) selectivity, though its pharmacokinetics and pharmacodynamics have been thoroughly characterized. The selected doses of both substances are comparable to those that elicit typical 5-HT_2C_-mediated behaviors in rats, such as hypophagia and nicotine self-administration (Higgins et al., 2013b, Thomsen et al., 2008). These two 5-HT_2C_R agonists induced a dose-dependent, marked and SB 242084-sensitive decrease of ASs up to 80 min post-injection. These results are consistent with, and extend, those obtained in WAG/Rij rats with the non-selective 5-HT_2/1B_R agonist mCPP (Jakus et al., 2003). The lack of pro-convulsant effect of the selective 5-HT_2C_ antagonist SB 242084 on ASs in GAERS, and in WAG/Rij rats (Jakus et al., 2003), might be surprising since 5-HT_2C_R KO mice show convulsive seizures and a higher susceptibility to different convulsive agents (Tecott et al., 1995). However, it is well know that the pathophysiological mechanisms leading to the expression of ASs are drastically different from those of convulsive seizures (Crunelli and Leresche, 2002). Conversely, in our hands SB 242084 had at some time-points a significant antiepileptic effect (limited to the total time spent in seizures). This convergence of anti-absence effects by 5-HT2CR agonists and antagonists, although surprising, has already been reported for their antidepressant effects (Di Giovanni and De Deurwaerdère, 2016, Millan, 2005).

The marked reduction in ASs elicited by intermediate doses of CP-809,101 and locarserin did not last for the entire 2 h observation period. This may be due to pharmacokinetics/pharmacodynamics features of the drugs, although the half-life of lorcaserin (>3 h) (Higgins et al., 2013b) makes this possibility unlikely (no data is available on CP-809,101 pharmacokinetics). Alternatively, the well-known, rapid (within minutes) 5-HT_2C_R desensitization, which however has been observed only *in vitro* however (Stout et al., 2002), might explain the relatively short duration of the anti-absence effect. Moreover, lorcaserin, but not CP-809,101, induced a drastic increase in seizure length. It is difficult at present to understand whether this contrasting effect may depend on the 5-HT_2C/2A_ selectivity of the two drugs, off-target effects and/or differences in their functional selectivity (Urban et al., 2007, Stroth and Svenningsson, 2012, Canal et al., 2013). Interestingly, the 5-HT_2C_R agonist RO60-0175 (3 mg/kg, i.p.) produces a similar anti-absence effect to that induced by CP-809,101, i.e., block of ASs without an increase in seizure length (unpublished observation).

### 4.3 Pathophysiological significance of 5-HT_2A_R- and 5-HT_2C_R-elicited block of ASs

5-HT fibers, originating from both dorsal and medial raphe nuclei provide a diffuse distribution in the cortico-thalamo-cortical circuit (Descarries et al., 2010), the key neuronal network responsible for AS generation (Crunelli and Leresche, 2002), with a preferential innervations of GABAergic cells in both brain regions (Hornung, 2010). In particular, 5-HT_2A_R levels are relatively high in the GABAergic neurons of the nucleus reticularis thalami (NRT) in rats (Bonnin et al., 2006, Rodriguez et al., 2011), particularly on their fine and medium-size dendrites (Aznar et al., 2003, Li et al., 2004, Rodriguez et al., 2011), but are also present to a lower level in sensory thalamic nuclei (Li et al., 2004), though apparently absent in mice dorsal lateral geniculate nucleus (dLGN) (Coulon et al., 2010). Moreover, 5-HT_2A_R and 5-HT_2C_R mRNA is detected in GABAergic interneurons isolated from the dLGN of young rats (Munsch et al., 2003). 5-HT_2A_Rs and 5-HT_2C_Rs are present in cortical GABAergic interneurons, and to a lesser extent in pyramidal neurons both in rats (Nocjar et al., 2015, Santana et al., 2004) and in primates (De Almeida and Mengod, 2007).

In view of this complex distribution of 5-HT_2A_Rs and 5-HT_2C_Rs in the cortico-thalamo-cortical system, and because of the lack of data on the cellular action of selective 5-HT_2A_R and 5-HT_2C_R agonists and antagonists on the neuronal components of this network, it is difficult to precisely relate the present findings on the modulation of ASs induced by systemic injection of 5-HT_2A_Rs and 5-HT_2C_Rs to known physiological effects of these ligands on different thalamic and cortical neuronal populations. Nevertheless, one might speculate that the putative 5-HT_2A_R-dependent i) decrease in firing of pyramidal cells *in vivo* (Ashby et al., 1990), ii) increase in IPSCs *in vitro* in pyramidal cells (Zhou and Hablitz, 1999) and iii) depolarization of fast-spiking interneurons *in vitro* (Weber and Andrade, 2010) may all be contributing to a reduced firing activity in the putative cortical “initiation site” from where SWDs firstly originate (Polack et al., 2007), thus explaining the reduction of ASs observed in the present study following 5-HT_2A_R activation. However, it should be noted that the putative 5-HT_2A_R-dependent increase in firing rate (Zhang and Arsenault, 2005) and burst discharges which have been reported in layer 5 pyramidal neurons *in vivo* (Celada et al., 2008, Spindle and Thomas, 2014) could be favoring, and not reducing, AS expression. At the thalamic level, 5-HT and putative 5-HT_2C_R agonists depolarize TC neurons (Chapin and Andrade, 2001, Meuth et al., 2006, Pape and McCormick, 1989). However, the α-methyl-5-HT-elicited excitation of TC neurons (Coulon et al., 2010) could be mediated by this drug activating 5-HT_7_Rs which are known to control the excitability of this brain region (Chapin and Andrade, 2001), and some TC neurons in higher order thalamic nuclei are hyperpolarized by 5-HT (Monckton and McCormick, 2002, Varela and Sherman, 2009). The depolarizing action on TC neurons, together with the putative 5-HT_2A/2C_R-mediated inhibition of NRT neuron burst firing (McCormick and Pape, 1990, McCormick and Wang, 1991), might counteract the increased tonic GABA_A_ inhibition of TC neurons in different absence models (Cope et al., 2009), thus contributing to the reduction in ASs by 5-HT_2A_R and 5-HT_2C_R agonists reported in the present study.

The possibility cannot be discarded, however, that the reduction in ASs observed in the present study following systemic injection of 5-HT_2A_R and 5-HT_2C_R agonists might result from an indirect action on other brain areas and/or physiological control systems. Firstly, since both 5-HT_2_R subtypes are well expressed in the basal ganglia (Li et al., 2004), which indirectly modulate AS generation (Deransart and Depaulis, 2002), the 5-HT_2A_R- and 5-HT_2C_R-induced reduction in ASs reported in the present study could be a consequence of changes in firing rate in these brain regions. Secondly, since ASs are less common during active wakefulness and non-REM sleep (Crunelli and Leresche, 2002, Depaulis and van Luijtelaar, 2006, Dewolfe et al., 2013), the 5-HT_2A_R- and 5-HT_2C_R-elicited decrease in ASs might be due to changes in wake/sleep states elicited by activation of these 5-HT receptor subtypes. Our study design did not allow us to robustly record circadian sleep which would have required acquiring a stable sleep baseline in GAERS before drug injection and a different habituation protocol. However we note that the available data on vigilance state alterations elicited by 5-HT_2A_R and 5-HT_2C_R activation are contradictory. 5-HT_2A_R and 5-HT_2C_R KO mice show increased waking and reduced non-REM sleep (Spindle and Thomas, 2014). On the other hand, systemic administration of DOI (Monti and Jantos, 2006) and RO60-0175 (Martin et al., 1998, Monti and Jantos, 2015) increases waking and reduces non-(rapid eye movement) REM and REM sleep. Opposite results have been obtained with selective 5-HT_2A_R agonist, and 5-HT_2C_R antagonists or non-selective 5-HT_2A/2C_R antagonists that increased drowsiness and non-REM sleep and reduced REM sleep (Morairty et al., 2008, Popa et al., 2005). Nevertheless, our analysis of the interictal EEG in the same GAERS rats where ASs were investigated indicate a decrease in gamma waves by TCB-2 and CP-809,101, a decrease in alpha waves by TCB-2 and locarserin, and an increase of delta waves by CP-809,101 and locarserin. Overall, therefore, these results provide indirect evidence that these drugs decrease vigilance and increase non-REM sleep, effects which might contribute to a reduction in ASs. Indeed, it is interesting to note that 5-HT_2A_R and 5-HT_2C_R activation produces similar actions on vigilance states and ASs whereas these two classes of 5-HTRs produce opposite effects on a plethora of other behaviors (Boulougouris and Robbins, 2010, Cunningham et al., 2013, Di Giovanni and De Deurwaerdère, 2016, Di Giovanni et al., 1999, Di Giovanni et al., 2008, Di Matteo et al., 2008, Fletcher et al., 2012, Halberstadt et al., 2009, Winstanley et al., 2004).

Another possibility that could be considered is that the stereotypic behaviors induced by 5-HT_2A_R and 5-HT_2C_R activation might contribute to the reduction in absence seizures observed in this study. Although a causal relationship between wet dog shakes, head twitches and penile grooming cannot be fully ruled out, these behaviours decrease and mostly disappear (our observation and (Halberstadt and Geyer, 2013)) 30 min after the 5-HT_2A/2C_R agonist administration while their antiabsence effect is of much longer duration (>1.5 h).

Finally, it is worth to note that the highest doses of the 5-HT_2A_R agonist TCB-2 and the 5-HT_2C_R agonist CP809101 produced a small increase in the peak SWD frequency, while both doses of the 5-HT_2A_R antagonist M100907 induced a small, but significant decrease in the peak SWD frequency. Although the mechanism that pace the rhythm of SWDs in not completely understood, it is known that the peak SWD frequency differs in various experimental models. In fact, gamma-hydroxybutyrate (GHB)- and PTZ-induced SWDs are generally in the range of 4–6 Hz in the rat (McLean et al., 2004, Venzi et al., 2015), while SWDs in genetic absence models, as GAERS, WAG/Rij and Long Evans rats, generally appear at frequencies of 7 Hz or higher (Coenen and Van Luijtelaar, 2003, Crunelli and Leresche, 2002, Shaw, 2004). Therefore, it is reasonable to assume that the rodent thalamortical network has the intrinsic ability of producing SWD oscillations at various frequencies and pharmacological interventions are capable of modulating the pace of this oscillation. Although there are few examples of this phenomenon, it was reported that systemic administration of carbamazepine reduced the frequency of PTZ-elicited SWDs by ∼0.5 Hz (McLean et al., 2004). Moreover, application of lidocaine on the perioral region of the somatosensory cortex was shown to shift the peak frequency of SWDs towards slower frequencies, although this effect was less pronounced when recorded in brain regions further away from the injection site (Sitnikova and van Luijtelaar, 2004). Clearly, the current lack of knowledge of the electrophysiological effects of 5-HT_2A_R and 5-HT_2C_R agonists and antagonist in cortical and thalamic neurons limits our ability to pinpoint a mechanism on the action of these compounds on SWD frequency. Future studies where local administration of drugs is coupled to single units extracellular recordings *in vivo* at the site of administration (Taylor et al., 2014) may help to elucidate this question.

### 4.4 Therapeutic potential of 5-HT_2A_R and 5-HT_2C_R ligands in ASs

The results reported here suggest that 5-HT_2A_Rs and 5-HT_2C_Rs might be potential targets for novel anti-absence drugs. However, the potential hallucinogenic activity of 5-HT_2A_R agonists must be taken into account, though 5-hydroxytryptophan, 5-HT_1A_R antagonists, benzodiazepines and cannabinoid (CB) antagonists/inverse agonists elicit head-twitch behavior, but lacks hallucinogenic effects in humans (Fantegrossi et al., 2005). Moreover, the activation of 5-HT_2A_R heteroreceptor complexes with mGluR2 (Gonzalez-Maeso et al., 2008) and D2Rs (Fuxe et al., 2014), CB1Rs (Vinals et al., 2015) and 5-HT_2C_Rs (Herrick-Davis et al., 2005) might be important for the effects observed here and for potential targets for drug development. Of note, our results with 5-HT_2A_R antagonists warn that atypical antipsychotics, which exert their therapeutic action, at least in part, by blocking 5-HT_2A_R-mediated responses (Meltzer, 1995) and some 5-HT_2A_R antagonists, which are being developed for the treatment of insomnia or anxiety, could potentially induce an increase in seizure length in patients with ASs. 5-HT_2C_R-targeting drugs, therefore, appear at present a safer and more promising avenue for novel anti-absence medicines, especially in view of the fact that lorcaserin has already been approved for human use. Moreover, since depression/anxiety-like symptoms are common comorbid psychiatric disorders both in pediatric epileptic patients (Vega et al., 2011) and animal models of absence epilepsy (Jones et al., 2008, Sarkisova and van Luijtelaar, 2012), the 5-HT_2C_R agonist antidepressant properties (Di Giovanni and De Deurwaerdère, 2016, Millan, 2005) make this receptor even a more attractable target for treatment of absence epilepsy. Nevertheless, the ability of some 5-HT_2C_R agonists to increase seizure length as shown in this study suggests caution.

## Conflict of interest

None.

## Author contribution

VC and GDiG designed research; MV, FD, VC and GDiG designed experiments; MV, FD, CB, JB and AC performed experiments and analyzed data; MV, VC and GDiG wrote the paper.

## Figures and Tables

**Table 1:** Selectivity profile of 5-HT_2A/2C_ drugs used in the study^i^.

Compound	5-HT_2A_			5-HT_2B_			5-HT_2c_			2C/2A
Ki (nM) a	pEC_50_^b^	Efficacy c	Ki (nM)	pEC_50_	Efficacy	Ki (nM)	pEC_50_	Efficacy
5-HT_2A_Rs										
TCB-2^d^	0.73	6.8	–	–	–	–	–	–	–	–
MDL11,939^e^	2.8	–	–	1419	–	–	853	–	–	–
M100,907^f^	1.9^e^	8.9	–	261^e^	6	–	88^e^	7.7	–	**0.06**
5-HT_2C_Rs										
CP-809,101^g^	6	6.8	0.67	64	7.2	0.57	1.6	10	0.93	**1585**
Lorcaserin^h^	159	6.7	1	190	6.0	1	29	7.9	1	**16**
SB242084^f^	851	6.8	–	45	7.0	–	7.0	9	–	**158**

a Ki: binding affinity.

b pEC50, potency: negative logarithm of the EC50 (half-maximal effective concentration).

c Efficacy relative to response to a supramaximal 5-HT concentration.

d (McLean et al., 2006).

e (Pehek et al., 2006).

f (Bromidge et al., 1997).

g (Siuciak et al., 2007).

h (Thomsen et al., 2008).

i Modified from (Higgins et al., 2013a).

## References

[bib1] Applegate C.D., Tecott L.H. (1998). Global increases in seizure susceptibility in mice lacking 5-HT2C receptors: a behavioral analysis. Exp. Neurol..

[bib105] Ashby C.R., Jiang L.H., Wang R.Y. (1990). Chronic brl-43694, a selective 5-ht3 receptor antagonist, fails to alter the number of spontaneously active midbrain dopamine neurons. Eur. J. Pharmacol..

[bib2] Aznar S., Qian Z., Shah R., Rahbek B., Knudsen G.M. (2003). The 5-HT1A serotonin receptor is located on calbindin- and parvalbumin-containing neurons in the rat brain. Brain Res..

[bib3] Bagdy G., Kecskemeti V., Riba P., Jakus R. (2007). Serotonin and epilepsy. J. Neurochem..

[bib4] Bedard P., Pycock C.J. (1977). “Wet-dog” shake behaviour in the rat: a possible quantitative model of central 5-hydroxytryptamine activity. Neuropharmacology.

[bib5] Bercovici E., Cortez M.A., Wang X., Snead O.C. (2006). Serotonin depletion attenuates AY-9944-mediated atypical absence seizures. Epilepsia.

[bib6] Bonnin A., Peng W., Hewlett W., Levitt P. (2006). Expression mapping of 5-HT1 serotonin receptor subtypes during fetal and early postnatal mouse forebrain development. Neuroscience.

[bib7] Bonnycastle D.D., Giarman N.J., Paasonen M.K. (1957). Anticonvulsant compounds and 5-hydroxytryptamine in rat brain. Br. J. Pharmacol. Chemother..

[bib8] Boulougouris V., Robbins T.W. (2010). Enhancement of spatial reversal learning by 5-HT2C receptor antagonism is neuroanatomically specific. J. Neurosci..

[bib9] Bromidge S.M., Duckworth M., Forbes I.T., Ham P., King F.D., Thewlis K.M., Blaney F.E., Naylor C.B., Blackburn T.P., Kennett G.A., Wood M.D., Clarke S.E. (1997). 6-Chloro-5-methyl-1-[[2-[(2-methyl-3-pyridyl)oxy]-5-pyridyl]carbamoyl]- indoline (SB-242084): the first selective and brain penetrant 5-HT2C receptor antagonist. J. Med. Chem..

[bib106] Canal C.E., Booth R.G., Morgan D. (2013). Support for 5-ht2c receptor functional selectivity in vivo utilizing structurally diverse, selective 5-ht2c receptor ligands and the 2,5-dimethoxy-4-iodoamphetamine elicited head-twitch response model. Neuropharmacology.

[bib10] Celada P., Puig M.V., Diaz-Mataix L., Artigas F. (2008). The hallucinogen DOI reduces low-frequency oscillations in rat prefrontal cortex: reversal by antipsychotic drugs. Biol. Psychiatry.

[bib11] Chapin E.M., Andrade R. (2001). A 5-HT(7) receptor-mediated depolarization in the anterodorsal thalamus. II. Involvement of the hyperpolarization-activated current I(h). J. Pharmacol. Exp. Ther..

[bib12] Citraro R., Leo A., De Fazio P., De Sarro G., Russo E. (2015). Antidepressants but not antipsychotics have antiepileptogenic effects with limited effects on comorbid depressive-like behaviour in the WAG/Rij rat model of absence epilepsy. Br. J. Pharmacol..

[bib13] Coenen A.M., Van Luijtelaar E.L. (2003). Genetic animal models for absence epilepsy: a review of the WAG/Rij strain of rats. Behav. Genet..

[bib14] Cope D.W., Di Giovanni G., Fyson S.J., Orban G., Errington A.C., Lorincz M.L., Gould T.M., Carter D.A., Crunelli V. (2009). Enhanced tonic GABAA inhibition in typical absence epilepsy. Nat. Med..

[bib15] Coulon P., Kanyshkova T., Broicher T., Munsch T., Wettschureck N., Seidenbecher T., Meuth S.G., Offermanns S., Pape H.C., Budde T. (2010). Activity modes in Thalamocortical relay neurons are modulated by g(q)/g(11) family g-proteins – serotonergic and glutamatergic signaling. Front. Cell Neurosci..

[bib16] Crunelli V., Leresche N. (2002). Childhood absence epilepsy: genes, channels, neurons and networks. Nat. Rev. Neurosci..

[bib17] Cunningham K.A., Anastasio N.C., Fox R.G., Stutz S.J., Bubar M.J., Swinford S.E., Watson C.S., Gilbertson S.R., Rice K.C., Rosenzweig-Lipson S., Moeller F.G. (2013). Synergism between a serotonin 5-HT2A receptor (5-HT2AR) antagonist and 5-HT2CR agonist suggests new pharmacotherapeutics for cocaine addiction. ACS Chem. Neurosci..

[bib18] Danober L., Deransart C., Depaulis A., Vergnes M., Marescaux C. (1998). Pathophysiological mechanisms of genetic absence epilepsy in the rat. Prog. Neurobiol..

[bib19] De Almeida J., Mengod G. (2007). Quantitative analysis of glutamatergic and GABAergic neurons expressing 5-HT2A receptors in human and monkey prefrontal cortex. J. Neurochem..

[bib20] Depaulis A., David O., Charpier S. (2016). The genetic absence epilepsy rat from Strasbourg as a model to decipher the neuronal and network mechanisms of generalized idiopathic epilepsies. J. Neurosci. Methods.

[bib21] Depaulis A., van Luijtelaar G., Moshé A.P.A.S.L. (2006). Chapter 18-Genetic models of absence epilepsy in the rat. Models of Seizures and Epilepsy.

[bib22] Deransart C., Depaulis A. (2002). The control of seizures by the basal ganglia? A review of experimental data. Epileptic Disord..

[bib23] Descarries L., Riad M., Parent M., Christian P.M.A.B.L.J. (2010). Ultrastructure of the serotonin innervation in the Mammalian central nervous system. Handbook of Behavioral Neuroscience.

[bib24] Dewolfe J.L., Malow B., Huguenard J., Stickgold R., Bourgeois B., Holmes G.L. (2013). Sleep and epilepsy: a summary of the 2011 merritt-putnam symposium. Epilepsy Curr..

[bib25] Di Giovanni G., De Deurwaerdère P. (2016). New therapeutic opportunities for 5-HT2C receptor ligands in neuropsychiatric disorders. Pharmacol. Ther..

[bib26] Di Giovanni G., De Deurwaerdere P., Di Mascio M., Di Matteo V., Esposito E., Spampinato U. (1999). Selective blockade of serotonin-2C/2B receptors enhances mesolimbic and mesostriatal dopaminergic function: a combined in vivo electrophysiological and microdialysis study. Neuroscience.

[bib27] Di Giovanni G., Di Matteo V., Pierucci M., Esposito E. (2008). Serotonin-dopamine interaction: electrophysiological evidence. Prog. Brain Res..

[bib28] Di Matteo V., Di Giovanni G., Esposito E. (2000). SB 242084: a selective 5-HT2C receptor antagonist. CNS Drug Rev..

[bib29] Di Matteo V., Di Giovanni G., Pierucci M., Esposito E. (2008). Serotonin control of central dopaminergic function: focus on in vivo microdialysis studies. Prog. Brain Res..

[bib30] Dudley M.W., Wiech N.L., Miller F.P., Carr A.A., Cheng H.C., Roebel L.E., Doherty N.S., Yamamura H.I., Ursillo R.C., Palfreyman M.G. (1988). Pharmacological effects of MDL 11,939: a selective, centrally acting antagonist of 5-HT2 receptors. Drug Dev. Res..

[bib31] Fantegrossi W.E., Ko M.C.H., Woods J.H., Richelson E. (2005). Antinociceptive, hypothermic, hypotensive, and reinforcing effects of a novel neurotensin receptor agonist, NT69L, in rhesus monkeys. Pharmacol. Biochem. Behav..

[bib32] FDA (2012). FDA Briefing Document NDA 22529 Lorcaserin Hydrochloride.

[bib33] Fletcher P.J., Grottick A.J., Higgins G.A. (2002). Differential effects of the 5-HT(2A) receptor antagonist M100907 and the 5-HT(2C) receptor antagonist SB242084 on cocaine-induced locomotor activity, cocaine self-administration and cocaine-induced reinstatement of responding. Neuropsychopharmacology.

[bib34] Fletcher P.J., Rizos Z., Noble K., Soko A.D., Silenieks L.B., Le A.D., Higgins G.A. (2012). Effects of the 5-HT2C receptor agonist Ro60-0175 and the 5-HT2A receptor antagonist M100907 on nicotine self-administration and reinstatement. Neuropharmacology.

[bib35] Fox M.A., French H.T., LaPorte J.L., Blackler A.R., Murphy D.L. (2010). The serotonin 5-HT(2A) receptor agonist TCB-2: a behavioral and neurophysiological analysis. Psychopharmacol. Berl..

[bib36] Fuxe K., Borroto-Escuela D.O., Romero-Fernandez W., Palkovits M., Tarakanov A.O., Ciruela F., Agnati L.F. (2014). Moonlighting proteins and protein-protein interactions as neurotherapeutic targets in the G protein-coupled receptor field. Neuropsychopharmacology.

[bib37] Gharedaghi M.H., Seyedabadi M., Ghia J.E., Dehpour A.R., Rahimian R. (2014). The role of different serotonin receptor subtypes in seizure susceptibility. Exp. Brain Res..

[bib38] Goda S.A., Piasecka J., Olszewski M., Kasicki S., Hunt M.J. (2013). Serotonergic hallucinogens differentially modify gamma and high frequency oscillations in the rat nucleus accumbens. Psychopharmacol. Berl..

[bib39] Gonzalez-Maeso J., Ang R.L., Yuen T., Chan P., Weisstaub N.V., Lopez-Gimenez J.F., Zhou M., Okawa Y., Callado L.F., Milligan G., Gingrich J.A., Filizola M., Meana J.J., Sealfon S.C. (2008). Identification of a serotonin/glutamate receptor complex implicated in psychosis. Nature.

[bib40] Guiard B.P., Di Giovanni G. (2015). Central Serotonin-2A (5-HT2A) receptor dysfunction in depression and epilepsy: The missing link?. Front. Pharmacol..

[bib41] Halberstadt A.L., Geyer M.A. (2013). Characterization of the head-twitch response induced by hallucinogens in mice: detection of the behavior based on the dynamics of head movement. Psychopharmacol. Berl..

[bib42] Halberstadt A.L., van der Heijden I., Ruderman M.A., Risbrough V.B., Gingrich J.A., Geyer M.A., Powell S.B. (2009). 5-HT(2A) and 5-HT(2C) receptors exert opposing effects on locomotor activity in mice. Neuropsychopharmacology.

[bib43] Hannon J., Hoyer D. (2008). Molecular biology of 5-HT receptors. Behav. Brain Res..

[bib44] Heisler L.K., Chu H.M., Tecott L.H. (1998). Epilepsy and obesity in serotonin 5-HT2C receptor mutant mice. Ann. N. Y. Acad. Sci..

[bib45] Herrick-Davis K., Grinde E., Harrigan T.J., Mazurkiewicz J.E. (2005). Inhibition of serotonin 5-hydroxytryptamine2C receptor function through heterodimerization: receptor dimers bind two molecules of ligand and one G-protein. J. Biol. Chem..

[bib46] Higgins G.A., Ouagazzal A.M., Grottick A.J. (2001). Influence of the 5-HT(2C) receptor antagonist SB242,084 on behaviour produced by the 5-HT(2) agonist Ro60-0175 and the indirect 5-HT agonist dexfenfluramine. Br. J. Pharmacol..

[bib47] Higgins G.A., Sellers E.M., Fletcher P.J. (2013). From obesity to substance abuse: therapeutic opportunities for 5-HT2C receptor agonists. Trends Pharmacol. Sci..

[bib48] Higgins G.A., Silenieks L.B., Lau W., de Lannoy I.A., Lee D.K., Izhakova J., Coen K., Le A.D., Fletcher P.J. (2013). Evaluation of chemically diverse 5-HT(2)c receptor agonists on behaviours motivated by food and nicotine and on side effect profiles. Psychopharmacol. Berl..

[bib49] Hornung J.-P., Christian P.M.A.B.L.J. (2010). The neuronatomy of the serotonergic system. Handbook of Behavioral Neuroscience.

[bib50] Hoyer D., Hannon J.P., Martin G.R. (2002). Molecular, pharmacological and functional diversity of 5-HT receptors. Pharmacol. Biochem. Behav..

[bib51] Iatsenko D., McClintock P.V.E., Stefanovska A. (2013). Linear and Synchrosqueezed Time-frequency Representations Revisited. Part II: Resolution, Reconstruction and Concentration. arxiv:1310.7274.

[bib52] Isaac M. (2005). Serotonergic 5-HT2C receptors as a potential therapeutic target for the design antiepileptic drugs. Curr. Top. Med. Chem..

[bib53] Jakus R., Bagdy G., Di Giovanni G., Esposito E., Di Matteo V. (2011). The role of 5-HT2C receptor in epilepsy. In: 5-HT2C receptors in the pathophysiology of CNS disease. 5-HT2C Receptors in the Pathophysiology of CNS Disease.

[bib54] Jakus R., Graf M., Juhasz G., Gerber K., Levay G., Halasz P., Bagdy G. (2003). 5-HT2C receptors inhibit and 5-HT1A receptors activate the generation of spike-wave discharges in a genetic rat model of absence epilepsy. Exp. Neurol..

[bib55] Jones N.C., Salzberg M.R., Kumar G., Couper A., Morris M.J., O'Brien T.J. (2008). Elevated anxiety and depressive-like behavior in a rat model of genetic generalized epilepsy suggesting common causation. Exp. Neurol..

[bib56] Kehne J.H., Baron B.M., Carr A.A., Chaney S.F., Elands J., Feldman D.J., Frank R.A., van Giersbergen P.L., McCloskey T.C., Johnson M.P., McCarty D.R., Poirot M., Senyah Y., Siegel B.W., Widmaier C. (1996). Preclinical characterization of the potential of the putative atypical antipsychotic MDL 100,907 as a potent 5-HT2A antagonist with a favorable CNS safety profile. J. Pharmacol. Exp. Ther..

[bib57] Kennett G.A., Wood M.D., Bright F., Trail B., Riley G., Holland V., Avenell K.Y., Stean T., Upton N., Bromidge S., Forbes I.T., Brown A.M., Middlemiss D.N., Blackburn T.P. (1997). SB 242084, a selective and brain penetrant 5-HT2C receptor antagonist. Neuropharmacology.

[bib58] Li Q.H., Nakadate K., Tanaka-Nakadate S., Nakatsuka D., Cui Y.L., Watanabe Y. (2004). Unique expression patterns of 5-HT2A and 5-HT2C receptors in the rat brain during postnatal development: Western blot and immunohistochemical analyses. J. Comp. Neurol..

[bib59] Lidster K., Jefferys J.G., Blumcke I., Crunelli V., Flecknell P., Frenguelli B.G., Gray W.P., Kaminski R., Pitkanen A., Ragan I., Shah M., Simonato M., Trevelyan A., Volk H., Walker M., Yates N., Prescott M.J. (2015). Opportunities for improving animal welfare in rodent models of epilepsy and seizures. J Neurosci Methods.

[bib60] Marescaux C., Vergnes M., Depaulis A. (1992). Genetic absence epilepsy in rats from Strasbourg–a review. J. Neural Transm. Suppl..

[bib61] Marescaux C., Vergnes M., Depaulis A. (1992). Neurotransmission in rats' spontaneous generalized nonconvulsive epilepsy. Epilepsy Res..

[bib62] Martin J.R., Bos M., Jenck F., Moreau J.L., Mutel V., Sleight A.J., Wichmann J., Andrews J.S., Berendsen H.H.G., Broekkamp C.L.E., Ruigt G.S.F., Kohler C., van Delft A.M.L. (1998). 5-HT2C receptor agonists: pharmacological characteristics and therapeutic potential. J. Pharmacol. Exp. Ther..

[bib63] McCormick D.A., Pape H.C. (1990). Properties of a hyperpolarization-activated cation current and its role in rhythmic oscillation in thalamic relay neurones. J. Physiol..

[bib64] McCormick D.A., Wang Z. (1991). Serotonin and noradrenaline excite GABAergic neurones of the guinea-pig and cat nucleus reticularis thalami. J. Physiol..

[bib65] McLean K.J., O'Brien T.J., Cook M.J., Vajda F.J. (2004). The influence of gender on the aggravation of absence seizures by carbamazepine in the low-dose pentylenetetrazol rat model. Seizure.

[bib66] McLean T.H., Parrish J.C., Braden M.R., Marona-Lewicka D., Gallardo-Godoy A., Nichols D.E. (2006). 1-Aminomethylbenzocycloalkanes: conformationally restricted hallucinogenic phenethylamine analogues as functionally selective 5-HT2A receptor agonists. J. Med. Chem..

[bib67] Meltzer H.Y. (1995). The role of serotonin in schizophrenia and the place of serotonin-dopamine antagonist antipsychotics. J. Clin. Psychopharmacol..

[bib68] Meuth S.G., Aller M.I., Munsch T., Schuhmacher T., Seidenbecher T., Meuth P., Kleinschnitz C., Pape H.C., Wiendl H., Wisden W., Budde T. (2006). The contribution of TWIK-related acid-sensitive K+-containing channels to the function of dorsal lateral geniculate thalamocortical relay neurons. Mol. Pharmacol..

[bib69] Millan M.J. (2005). Serotonin 5-HT2C receptors as a target for the treatment of depressive and anxious states: focus on novel therapeutic strategies. Therapie.

[bib70] Monckton J.E., McCormick D.A. (2002). Neuromodulatory role of serotonin in the Ferret Thalamus. J. Neurophysiol..

[bib71] Monti J.M., Jantos H. (2006). Effects of the serotonin 5-HT2A/2C receptor agonist DOI and of the selective 5-HT2A or 5-HT2C receptor antagonists EMD 281014 and SB-243213, respectively, on sleep and waking in the rat. Eur. J. Pharmacol..

[bib72] Monti J.M., Jantos H. (2015). The effects of systemic administration and local microinjection into the central nervous system of the selective serotonin 5-HT2C receptor agonist RO-600175 on sleep and wakefulness in the rat. Behav. Pharmacol..

[bib73] Morairty S.R., Hedley L., Flores J., Martin R., Kilduff T.S. (2008). Selective 5HT2A and 5HT6 receptor antagonists promote sleep in rats. Sleep.

[bib74] Munsch T., Freichel M., Flockerzi V., Pape H.C. (2003). Contribution of transient receptor potential channels to the control of GABA release from dendrites. Proc. Natl. Acad. Sci. U. S. A..

[bib75] Nichols D.E. (2004). Hallucinogens. Pharmacol. Ther..

[bib76] Nocjar C., Alex K.D., Sonneborn A., Abbas A.I., Roth B.L., Pehek E.A. (2015). Serotonin-2C and -2a receptor co-expression on cells in the rat medial prefrontal cortex. Neuroscience.

[bib77] Ohno Y., Sofue N., Imaoku T., Morishita E., Kumafuji K., Sasa M., Serikawa T. (2010). Serotonergic modulation of absence-like seizures in groggy rats: a novel rat model of absence epilepsy. J. Pharmacol. Sci..

[bib107] Oostenveld R., Fries P., Maris E., Schoffelen J.M. (2011). FieldTrip: open source software for advanced analysis of MEG, EEG, and invasive electrophysiological data. Comput. Intell. Neurosci..

[bib78] Orban G., Bombardi C., Marino Gammazza A., Colangeli R., Pierucci M., Pomara C., Pessia M., Bucchieri F., Benigno A., Smolders I., De Deurwaerdere P., Di Giovanni G. (2014). Role(s) of the 5-HT2C receptor in the development of maximal dentate activation in the hippocampus of anesthetized rats. CNS Neurosci. Ther..

[bib79] Pape H.C., McCormick D.A. (1989). Noradrenaline and serotonin selectively modulate thalamic burst firing by enhancing a hyperpolarization-activated cation current. Nature.

[bib80] Pehek E.A., Nocjar C., Roth B.L., Byrd T.A., Mabrouk O.S. (2006). Evidence for the preferential involvement of 5-HT2A serotonin receptors in stress- and drug-induced dopamine release in the rat medial prefrontal cortex. Neuropsychopharmacology.

[bib81] Polack P.O., Guillemain I., Hu E., Deransart C., Depaulis A., Charpier S. (2007). Deep layer somatosensory cortical neurons initiate spike-and-wave discharges in a genetic model of absence seizures. J. Neurosci..

[bib82] Popa D., Lena C., Fabre V., Prenat C., Gingrich J., Escourrou P., Hamon M., Adrien J. (2005). Contribution of 5-HT2 receptor subtypes to sleep-wakefulness and respiratory control, and functional adaptations in knock-out mice lacking 5-HT2A receptors. J. Neurosci..

[bib83] Richtand N.M., Welge J.A., Logue A.D., Keck P.E., Strakowski S.M., McNamara R.K. (2007). Dopamine and serotonin receptor binding and antipsychotic efficacy. Neuropsychopharmacology.

[bib84] Rodriguez J.J., Noristani H.N., Hoover W.B., Linley S.B., Vertes R.P. (2011). Serotonergic projections and serotonin receptor expression in the reticular nucleus of the thalamus in the rat. Synapse.

[bib85] Roth B.L. (2011). Irving Page Lecture: 5-HT(2A) serotonin receptor biology: interacting proteins, kinases and paradoxical regulation. Neuropharmacology.

[bib86] Santana N., Bortolozzi A., Serrats J., Mengod G., Artigas F. (2004). Expression of serotonin1A and serotonin2A receptors in pyramidal and GABAergic neurons of the rat prefrontal cortex. Cereb. Cortex.

[bib87] Sarkisova K., van Luijtelaar G. (2012). The WAG/Rij strain: a genetic animal model of absence epilepsy with comorbidity of depression. Prog. Neuropsychopharmacol. Biol. Psychiatry.

[bib88] Shaw F.Z. (2004). Is spontaneous high-voltage rhythmic spike discharge in Long Evans rats an absence-like seizure activity?. J. Neurophysiol..

[bib89] Sitnikova E., van Luijtelaar G. (2004). Cortical control of generalized absence seizures: effect of lidocaine applied to the somatosensory cortex in WAG/Rij rats. Brain Res..

[bib90] Siuciak J.A., Chapin D.S., McCarthy S.A., Guanowsky V., Brown J., Chiang P., Marala R., Patterson T., Seymour P.A., Swick A., Iredale P.A. (2007). CP-809,101, a selective 5-HT2C agonist, shows activity in animal models of antipsychotic activity. Neuropharmacology.

[bib91] Spindle M.S., Thomas M.P. (2014). Activation of 5-HT2A receptors by TCB-2 induces recurrent oscillatory burst discharge in layer 5 pyramidal neurons of the mPFC in vitro. Physiol. Rep..

[bib92] Stout B.D., Clarke W.P., Berg K.A. (2002). Rapid desensitization of the serotonin(2C) receptor system: effector pathway and agonist dependence. J. Pharmacol. Exp. Ther..

[bib108] Stroth N., Svenningsson P. (2012). Ligand-specific differential regulation of 5-hydroxytryptamine receptors: functional selectivity in serotonergic signaling. Wiley Interdiscip. Rev. Membr. Transp. Signal..

[bib93] Taylor H., Schmiedt J.T., Carcak N., Onat F., Di Giovanni G., Lambert R., Leresche N., Crunelli V., David F. (2014). Investigating local and long-range neuronal network dynamics by simultaneous optogenetics, reverse microdialysis and silicon probe recordings in vivo. J. Neurosci. Methods.

[bib94] Tecott L.H., Sun L.M., Akana S.F., Strack A.M., Lowenstein D.H., Dallman M.F., Julius D. (1995). Eating disorder and epilepsy in mice lacking 5-HT2c serotonin receptors. Nature.

[bib95] Thomsen W.J., Grottick A.J., Menzaghi F., Reyes-Saldana H., Espitia S., Yuskin D., Whelan K., Martin M., Morgan M., Chen W., Al-Shamma H., Smith B., Chalmers D., Behan D. (2008). Lorcaserin, a novel selective human 5-hydroxytryptamine2C agonist: in vitro and in vivo pharmacological characterization. J. Pharmacol. Exp. Ther..

[bib96] Tokuda S., Kuramoto T., Tanaka K., Kaneko S., Takeuchi I.K., Sasa M., Serikawa T. (2007). The ataxic groggy rat has a missense mutation in the P/Q-type voltage-gated Ca2+ channel alpha1A subunit gene and exhibits absence seizures. Brain Res..

[bib97] Upton N., Stean T., Middlemiss D., Blackburn T., Kennett G. (1998). Studies on the role of 5-HT2C and 5-HT2B receptors in regulating generalised seizure threshold in rodents. Eur. J. Pharmacol..

[bib109] Urban J.D., Clarke W.P., von Zastrow M., Nichols D.E., Kobilka B., Weinstein H., Javitch J.A., Roth B.L., Christopoulos A., Sexton P.M., Miller K.J., Spedding M., Mailman R.B. (2007). Functional selectivity and classical concepts of quantitative pharmacology. J. Pharmacol. Exp. Ther..

[bib98] Varela C., Sherman S.M. (2009). Differences in response to serotonergic activation between first and higher order Thalamic nuclei. Cereb. Cortex.

[bib99] Vega C., Guo J., Killory B., Danielson N., Vestal M., Berman R., Martin L., Gonzalez J.L., Blumenfeld H., Spann M.N. (2011). Symptoms of anxiety and depression in childhood absence epilepsy. Epilepsia.

[bib100] Venzi M., Di Giovanni G., Crunelli V. (2015). A critical evaluation of the gamma-hydroxybutyrate (GHB) model of absence seizures. CNS Neurosci. Ther..

[bib101] Vinals X., Moreno E., Lanfumey L., Cordomi A., Pastor A., de La Torre R., Gasperini P., Navarro G., Howell L.A., Pardo L., Lluis C., Canela E.I., McCormick P.J., Maldonado R., Robledo P. (2015). Cognitive impairment induced by delta9-tetrahydrocannabinol occurs through heteromers between cannabinoid CB1 and serotonin 5-HT2A receptors. PLoS Biol..

[bib102] Winstanley C.A., Theobald D.E., Dalley J.W., Glennon J.C., Robbins T.W. (2004). 5-HT2A and 5-HT2C receptor antagonists have opposing effects on a measure of impulsivity: interactions with global 5-HT depletion. Psychopharmacol. Berl..

[bib103] Zhang Z.-w., Arsenault D. (2005). Gain modulation by serotonin in pyramidal neurones of the rat prefrontal cortex. J. Physiol..

[bib104] Zhou F.M., Hablitz J.J. (1999). Activation of serotonin receptors modulates synaptic transmission in rat cerebral cortex. J. Neurophysiol..

